# Global trends and frontiers of research on pathologic myopia since the millennium: A bibliometric analysis

**DOI:** 10.3389/fpubh.2022.1047787

**Published:** 2022-12-06

**Authors:** Jingyuan Yang, Shan Wu, Chenxi Zhang, Weihong Yu, Rongping Dai, Youxin Chen

**Affiliations:** ^1^Department of Ophthalmology, Peking Union Medical College Hospital, Chinese Academy of Medical Sciences, and Peking Union Medical College, Beijing, China; ^2^Key Laboratory of Ocular Fundus Diseases, Chinese Academy of Medical Sciences, and Peking Union Medical College, Beijing, China; ^3^Department of Anaesthesiology, Beijing Hospital, National Center of Gerontology, Institute of Geriatric Medicine, Chinese Academy of Medical Sciences, Beijing, China

**Keywords:** pathologic myopia, bibliometric analysis, maculopathy, myopic degeneration, myopia control

## Abstract

**Background and purpose:**

Pathologic myopia (PM) is an international public health issue. This study aimed to analyze PM research trends by reporting on publication trends since 2000 and identifying influential journals, countries, authors, and keywords involved in PM.

**Methods:**

A bibliometric analysis was performed to evaluate global production and development trends in PM since 2000 and the keywords associated with PM.

**Results:**

A total of 1,435 publications were retrieved. PM has become a fascinating topic (with relative research interest ranging from 0.0018% in 2000 to 0.0044% in 2021) and a global public health issue. The top three countries with the highest number of publications were China, the USA, and Japan. The journals, authors, and institutions that published the most relevant literature came from these three countries. China exhibited the most rapid increase in the number of publications (from 0 in 2000 to 69 in 2021). *Retina* published the most papers on PM. Kyoko Ohno-Matsui and Tokyo Medical and Dental University contributed the most publications among authors and institutions, respectively. Based on keyword analysis, previous research emphasized myopic choroidal neovascularization and treatment, while recent hotspots include PM changes based on multimodal imaging, treatment, and pathogenesis. Keyword analysis also revealed that deep learning was the latest hotspot and has been used for the detection of PM.

**Conclusion:**

Our results can help researchers understand the current status and future trends of PM. China, the USA, and Japan have the greatest influence, based on the number of publications, top journals, authors, and institutions. Current research on PM highlights the pathogenesis and application of novel technologies, including multimodal imaging and artificial intelligence.

## Introduction

Myopia is a leading cause of vision loss worldwide, with an increasing trend; it can be divided into simple myopia and pathologic myopia (PM). There is no standardized definition for PM. However, most researchers and clinicians agree that PM is a subtype of myopia that accompanies characteristic myopic fundus changes that usually occur in eyes with high myopia, including myopic maculopathy equal to or more serious than diffuse choroidal atrophy or the presence of posterior staphyloma (1). The prevalence of myopic retinopathy in PM was reported to range from 1.2 to 3.7% in the population (2–6). Moreover, the visual prognosis for eyes with myopic maculopathy is much worse than that for those without maculopathy (7–9). A bibliometric analysis of publications is important due to the growing number of pathologic myopic eyes and their influence on public health, which has prompted researchers to analyze the development trend and research hotspots of PM.

Bibliometric analyses were first proposed in 1922 by Hulme; however (10), these analyses are not equal to reviews. Bibliometric analyses provide an overview of publications using mathematical and statistical methods and predict possible development trends based on citation reports and academic outputs. Bibliometric analysis, which has a century-long history, has been applied in medicine to investigate its development and trends.

Research on PM has received increasing attention in recent years, which makes it difficult for many researchers and clinicians to determine the research focus and frontiers of PM from the growing number of publications. However, to our knowledge, no bibliometric analysis has specifically focused on PM. Therefore, the current study aimed to investigate frontier research and PM trends across the international scientific literature based on the Web of Science (WOS) Core Collection over the past two decades. We also aimed to predict trends for the next few years, noting that the increase in the number of publications on PM is expected to lead to a valuable reference for clinicians and researchers.

## Methods

### Search strategy

The WOS Core Collection is the most suitable database for bibliometric analysis, particularly because of the high quality of the included publications. The search for papers to be included in the current study was carried out on 7 August 2022, and all the included publications were published from 1 January 2000, to 1 August 2022. The search strategy was “TS = pathologic myopia OR TS = myopic degeneration OR TS = myopic maculopathy.” One thousand and thirty-seven pieces of literature were identified. Two published poems and news items were excluded according to the document type. One thousand and thirty-five publications were finally included.

### Data collection

All the data were extracted and downloaded from the WOS Core Collection databases, including metrics of publication numbers, countries and regions, authors, citations, self-citations, and H-indexes. We also investigated the relationship between the global productivity of PM and the human development index (HDI), which measures the level of human development based on knowledge, life expectancy, and income per capita indicators rather than economic growth alone. The United Nations Development Programme published the Human Development Report 2020, in which countries and areas were divided into four categories based on HDI: very high human development, high human development, medium human development, and low human development (11). Countries and regions were classified according to the default classification in the WOS and the HDI. Prism 9, R (R. app. GUI 1.79) and its tools Biblioshiny, VOSviewer 1.6.18, and SPSS 26 were used to input and analyze the data.

### Bibliometric analysis

Descriptive indices were extracted from the WOS Core Collection and calculated using SPSS. The co-occurrence network was constructed using the VOSviewer software. Keywords were extracted from the titles and abstracts of the included publications. R and its tool Biblioshiny were used to generate word-cloud maps. To provide a better classification of keyword clusters and a better summarization of research trends, frequencies over 30 were the criteria for the inclusion of these analyses. H-indices were collected from the WOS database and can partially reflect the impact of researchers. Relative research interest (RRI) was measured as the number of publications in a specific field divided by the number of all publications in all fields. The value of this metric reflects the global attention and interest in a specific field. Higher RRI values represent more research interest and hotspots in this field. A third-order polynomial method was used in the prediction model using the Prism software. The average year of appearance was used to assess the novelty of the keywords.

## Results

### Contributions of various countries and regions to global publications

**Figure 1:**
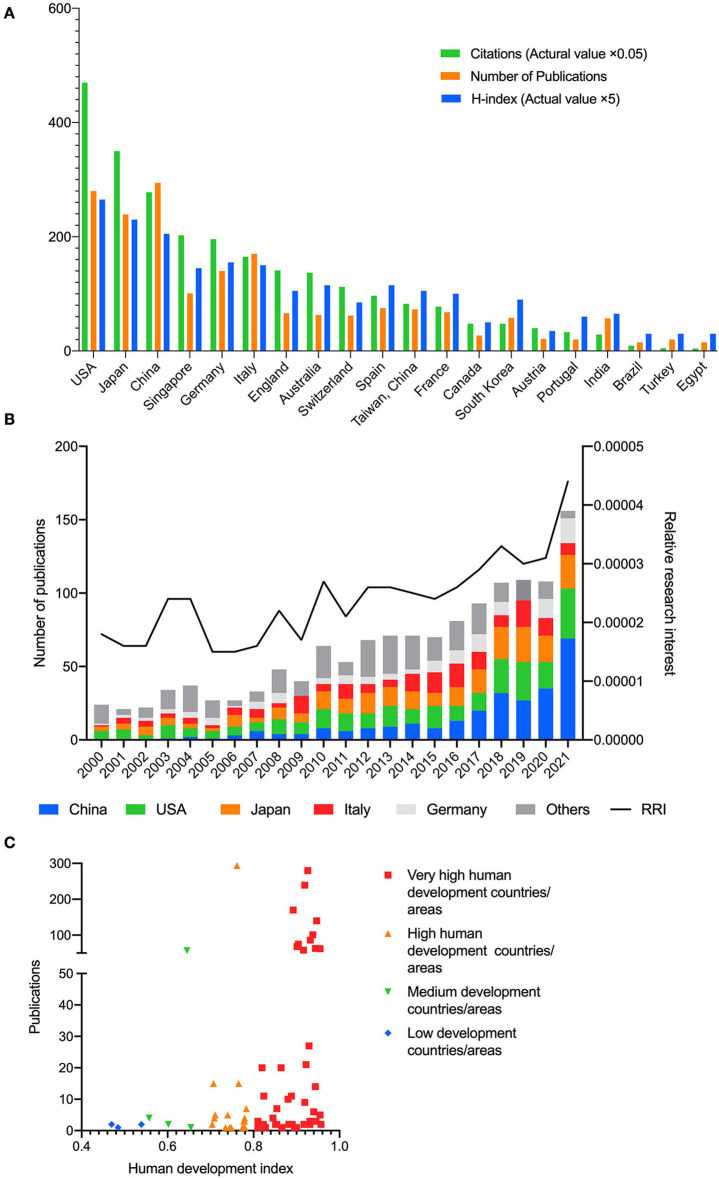
**(A)** Top 20 countries/regions in the publications on pathologic myopia. The green bar shows the number of citations (actual value multiply by 0.05), the orange bar shows the number of publications, and the blue bar shows the H-index (actual value multiply by 5). **(B)** The proportion of publications of China Mainland, USA, Japan, Italy, Germany, and other countries/regions and relative research interest (RRI) in each year on the field of pathologic myopia. **(C)** The number of publications on pathologic myopia in countries or areas of various levels of human development. Very high human development countries or areas contributed to most publications.

A total of 1,435 publications were analyzed. Since 2000, Mainland China has contributed to the majority of publications (294, 20.5%), followed by the USA (280, 19.5%) and Japan (239, 16.7%) (Figure 1A). The remaining countries had published fewer than 200 publications. The USA, Japan, China, Germany, Italy, and Singapore were the top five countries with the highest number of publications and H-index. The total number of publications on PM has grown over the past 22 years, especially in recent years (Figure 1B). Additionally, the RRI of PM has increased from 0.0018% in 2000 to 0.0044% in 2021, indicating that research interest in PM has continued to increase worldwide over the past two decades. In the first 8 months of 2022, the RRI of PM reaches 0.0046%. According to the HDI category, we noticed that most publications were from very high HDI countries or regions, and the numbers of publications on PM were consistent with the HDI classification (Figure 1C).

We analyzed the co-occurrence of 34 countries and regions (Supplementary Figure 1). The analysis suggests six clusters: (1) Mainland China, Australia, and Iran; (2) USA, Switzerland, France, South Korea, Canada, Austria, Brazil, Turkey, Greece, and Venezuela; (3) Japan, Singapore, Spain, and Portugal; (4) Germany, England, the Netherlands, Russia, Scotland, Northern Ireland, Poland, Wales, Ireland, and Egypt; (5) Italy, India, Tunisia, and Israel; and (6) Taiwan, Denmark, and Saudi Arabia.

**Figure 2:**
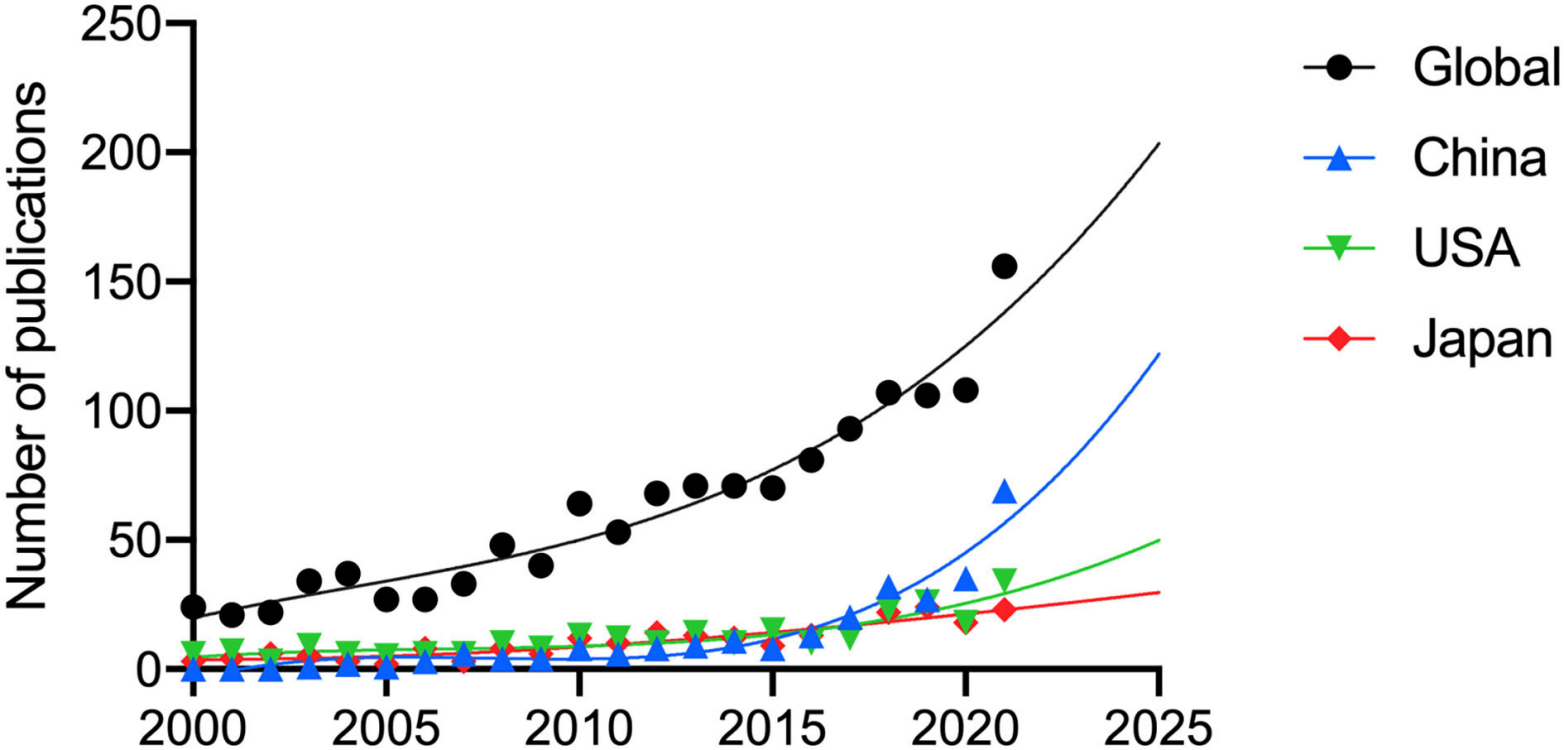
The publication trends and prediction curve of global and countries which had the most publications of pathologic myopia since 2000. **(A)** Global. **(B)** China Mainland. **(C)** USA. **(D)** Japan.

The publication rate of papers on PM has been increasing in the past two decades, and predictions for the coming years reflect this increase (Figure 2). China exhibited the most rapid increase in the number of publications in the last 5 years. China is also projected to maintain its leading position and show steady growth in the number of publications.

### Citations and H-index

WOS citation reports revealed a total of 21,432 citations without self-citations of the 30,234 relevant citations since 2000 (details for top countries and regions in Supplementary Table 1). Each paper cited an average of 21.07 times. The USA contributed the most citations (9,395 citations, 9,062 without self-citations) and the highest H-index (53) (Figure 1A) from 2000. Japan ranked second in terms of both citations (6,999 citations, 6,026 without self-citations) and H-index (46), and Mainland China ranked third in both citations (5,559 citations, 4,895 without self-citations) and H-index (41). The most-cited publication was cited 618 times. We divided the publications into three groups according to citation frequency: high frequency (more than 100 citations), medium frequency (>50 and <100 citations), and low frequency (<50 citations). Most publications were in a low-frequency group. One hundred and ten publications were cited with a medium frequency, and 46 were cited with a high frequency.

To investigate the distribution of citation numbers each year, we drew heat maps of each group of citation frequency (Supplementary Figures 2B–D). Every row in the heatmap represents a publication, the x-axis represents the year, and the color represents the citation number. The timespan of citations with high and medium frequency is similar and longer than most publications with low citation frequency. Moreover, most publications with high and medium citation frequencies have been published in the last 10 years (Supplementary Figure 2A).

### The leading institutions, journals, and authors

**Figure 3:**
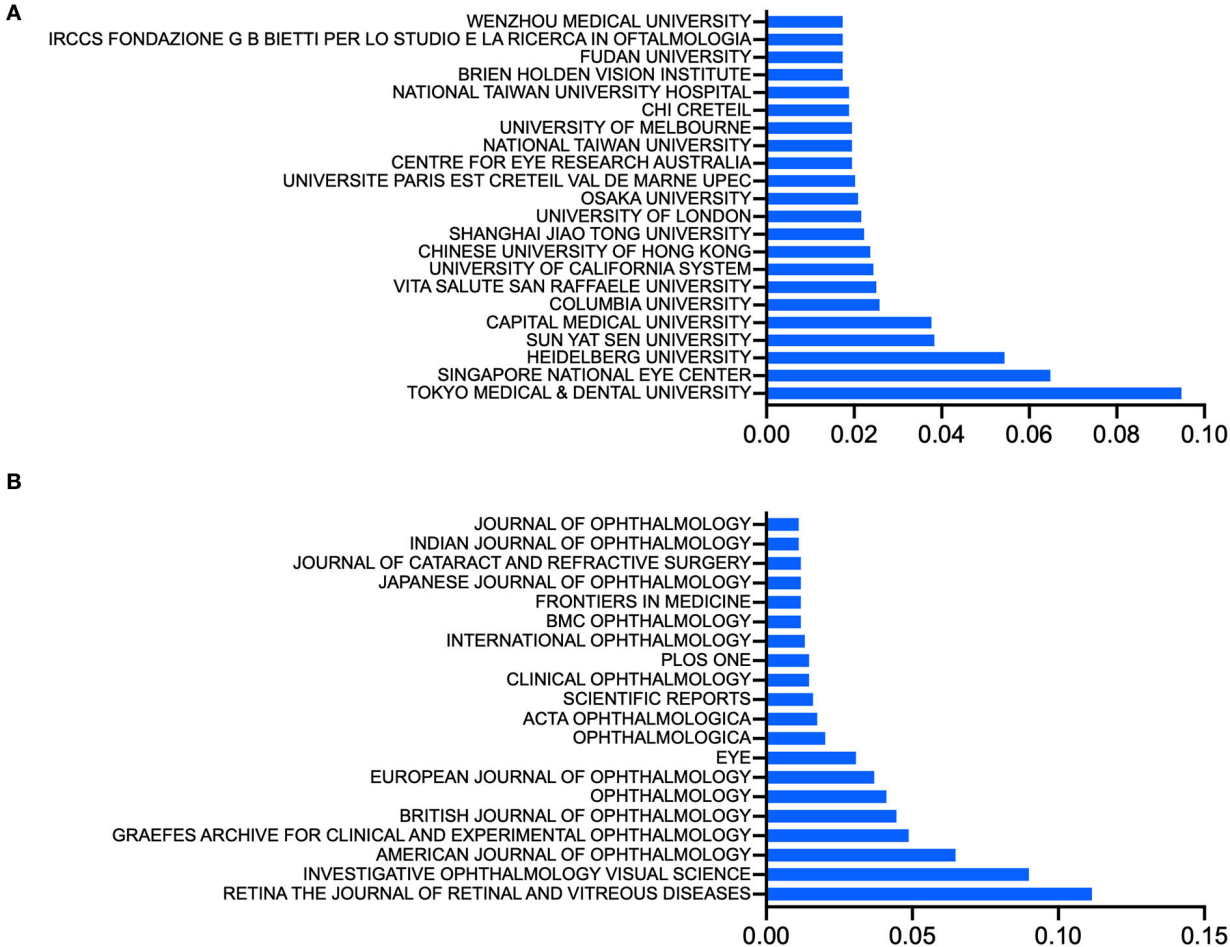
**(A)** Top institutions, ranked by the ratio of the number of publications from an institution to the total number of included publications about pathologic myopia. **(B)** Top journals, ranked by the ratio of the number of publications from a journal to the total number of included publications about pathologic myopia.

We investigated the top institutions in this field; the Tokyo Medical and Dental University in Japan (136, 9.5%), Singapore National Eye Center in Singapore (93, 6.5%), Ruprecht-Karls-Universität Heidelberg in Germany (78, 5.4%), and Sun Yat-Sen University (55, 3.8%) and Capital Medical University (54, 3.8%) in China published the most publications (Figure 3A).

Approximately half (726, 50.6%) of the papers on PM were published in 10 journals, including *Retina*, which published the maximum number of relevant publications (160). *Investigative Ophthalmology and Visual Science* and the *American Journal of Ophthalmology* published the second and third-most publications, with 129 and 93, respectively (Figure 3B).

The 10 papers with the most citations are listed in Table 1. The most cited paper was published in *Ophthalmic and Physiological Optics*, an ophthalmic periodical that has ceased publication, and was called *Myopia and Associated Pathological Complications*. The corresponding author was Seang Mei Saw. Most publications on PM were published in ophthalmology journals (Table 2).

**Table 1:** Top 10 papers with the most citations relevant to pathologic myopia.

**Title**	**Corresponding authors**	**Journal**	**Publication year**	**Total citations**
Myopia and associated pathological complications	Saw, SM	OPHTHALMIC AND PHYSIOLOGICAL OPTICS	2005	618
Enhanced depth imaging optical coherence tomography of the choroid in highly myopic eyes	Spaide, Richard F	AMERICAN JOURNAL OF OPHTHALMOLOGY	2009	572
Photodynamic therapy of subfoveal choroidal neovascularization in pathologic myopia with verteporfin-−1-year results of a randomized clinical trial—VIP report no. 1	Bressler, NM	OPHTHALMOLOGY	2001	377
The complex interactions of retinal, optical, and environmental factors in myopia etiology	Flitcroft, D. I	PROGRESS IN RETINAL AND EYE RESEARCH	2012	361
Prevalence and causes of low vision and blindness in a Japanese adult population—The Tajimi Study	Araie, Makoto	OPHTHALMOLOGY	2006	349
Epidemiology and disease burden of pathologic myopia and myopic choroidal neovascularization: an evidence-based systematic review	Wong, Tien Y	AMERICAN JOURNAL OF OPHTHALMOLOGY	2014	334
International photographic classification and grading system for myopic maculopathy	Ohno-Matsui, Kyoko	AMERICAN JOURNAL OF OPHTHALMOLOGY	2015	327
Corneal collagen crosslinking with riboflavin and ultraviolet A to treat induced keratectasia after laser in situ keratomileusis	Hafezi, Farhad	JOURNAL OF CATARACT AND REFRACTIVE SURGERY	2007	316
Efficacy of a deep learning system for detecting glaucomatous optic neuropathy based on color fundus photographs	He, Mingguang	OPHTHALMOLOGY	2018	292
Long-term pattern of progression of myopic maculopathy: a natural history study	Ohno-Matsui, Kyoko	OPHTHALMOLOGY	2010	292

**Table 2:** Top 10 Web of Science categories of journals on pathologic myopia research.

**Web of science categories**	**No. of publications (%)**
Ophthalmology	1,189 (82.86)
Medicine general internal	76 (5.3)
Surgery	53 (3.69)
Multidisciplinary sciences	47 (3.28)
Pharmacology pharmacy	33 (2.3)
Medicine research experimental	29 (2.02)
Genetics heredity	15 (1.05)
Optics	14 (0.98)
Biochemistry molecular biology	13 (0.91)
Engineering biomedical	12 (0.84)

The top 10 authors in this field are listed in Table 3 according to the number of publications. The works of Kyoko Ohno-Matsui from Tokyo Medical and Dental University have been published the most since 2000, with 139 papers and 4,710 citations (4,033 without self-citations). The H-indexes of both the author and the institution were 39. Jost B. Jonas, from Ruprecht-Karls-Universität Heidelberg, ranked second, with 77 publications and 2,224 citations (2,025 without self-citations). Tien Y Wong ranked third with 44 publications and 2,135 citations (2,045 without self-citations) (Table 3).

**Table 3:** Top 10 authors who published most in the field of pathologic myopia.

**Author**	**Country**	**Latest affiliation**	**No. of publications**	**No. of citations**
Kyoko Ohno-Matsui	Japan	Tokyo Medical and Dental University	139	4,710
Jost B Jonas	Germany	Ruprecht-Karls-Universität Heidelberg	77	2,224
Tien Y Wong	Singapore	National University of Singapore	44	2,135
Seang Mei Saw	Singapore	Singapore National Eye Center	42	2,088
Muka Moriyama	Japan	Tokyo Medical and Dental University	41	1,931
Noriaki Shimada	Japan	Kurashiki Central Hospital	35	1,588
Takeshi Yoshida	Japan	Kyoto University	35	1,290
Francesco Bandello	Italy	Università Vita-Salute San Raffaele	34	903
Quan V.Hoang	Singapore	National University of Singapore	34	229
Chee Wai Wong	Singapore	National University of Singapore	33	228

We also analyzed the cooperation between investigators (Supplementary Figure 3). The node size within a collaboration network indicates the strength of the connections between each author. Several authors, including Kyoko Ohno-Matsui, Takeshi Yoshida, Muka Moriyama, Jost B. Jonas, and Noriaki Shimada, closely cooperated with other researchers and teams.

### Research hotspots in PM

**Figure 4:**
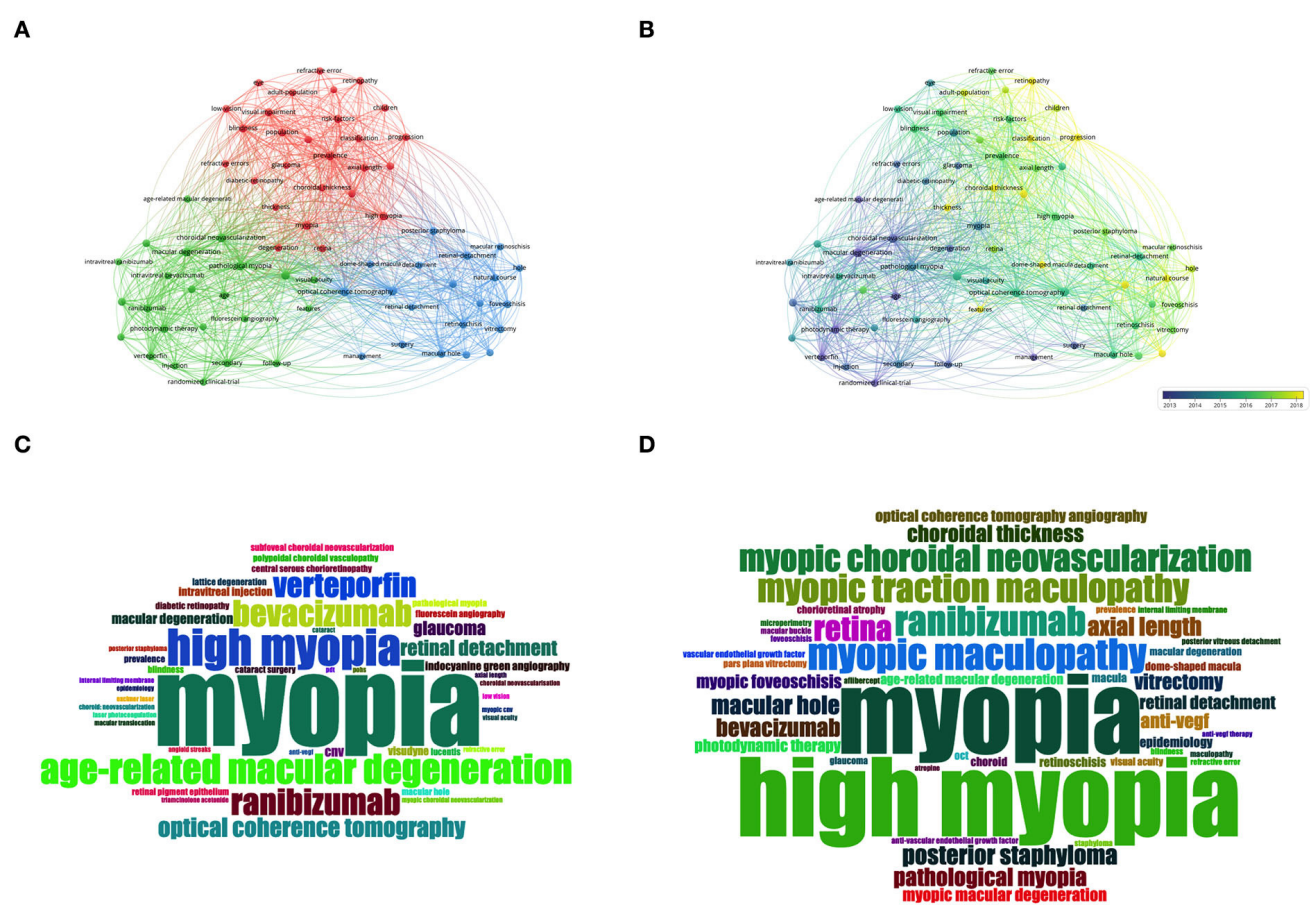
Keywords analysis by VOSviewer and R. **(A)** Co-occurrence map of keywords in titles and abstracts. Keywords were classified into 3 clusters by co-occurrence frequency, including an epidemiology-related cluster (in red), a treatment-related cluster (in green), and a lesion-related cluster (in blue). **(B)** Color-coded map of keywords by the average time of appearance. Blue keywords appeared earlier, while yellow keywords appeared more recently. **(C)** The word-cloud map of the most frequent keywords from 2000 to 2010. **(D)** The word-cloud map of the most frequent keywords from 2011 to August 2022.

Keyword analysis identified the most frequently used words and their linkages within the field of PM research. We analyzed keywords that appeared more than 30 times across the included publications. Merging repeated words, excluding meaningless ones, resulted in 70 keywords that could be divided into three primary clusters by co-occurrence frequency, including an epidemiology-related cluster (in red), a treatment-related cluster (in green), and a lesion-related cluster (in blue) (Figure 4A). Keywords with high link strength were assigned to the same cluster.

We also color-coded the keywords by the average time of appearance and found that most of the keywords appeared in recent years (Figure 4B). Figure 4B shows the temporal evolution of these keywords. The *classification* and *progression* keywords were the latest keywords with high occurrence.

To attain an intuitive impression of the most frequent keywords, we listed the most used keywords in 2000–2010 and 2011–2022 in word-cloud images, respectively (Figure 4C for keywords from 2000 to 2010 and Figure 4D for keywords from 2011 to August 2022). Besides the keywords of myopia and high myopia, keywords related to pathologic lesions and treatments, such as myopic maculopathy, myopic choroidal neovascularization (mCNV), and ranibizumab, were the most frequent words. We extracted the most frequent keywords to explore changes in hotspots and keywords in the field (Supplementary Figure 4). A changing trend was noticed from treatment for choroidal neovascularization to myopic lesions and complications and to deep learning.

## Discussion

This study involved a bibliometric analysis focusing on PM over the last two decades. We identified an increasing trend in publications on PM since 2000 (from 24 publications in 2000 to 156 publications in 2021). Although China and the USA had the most publications (294 and 280, respectively), authors from Japan, Singapore, and Germany had the greatest influence (4, 4, and 1 of the top 10 authors, respectively). To our knowledge, this is the first bibliometric analysis to focus on PM.

### Country- or region-based contributions to PM research

The prevalence of PM in a country or region did not completely parallel its number of publications (Table 4). For example, although the USA contributed a large number of PM publications, only Asian and Pacific Islanders in the USA had a greater prevalence (ethnicity OR 1.64) (12). Conversely, Asian countries, especially some countries in East Asia, including Japan and China (1.7% and 3.7%, respectively), had a relatively higher prevalence of myopic retinopathy than some Western countries, such as Australia (1.2%) (2–6). Therefore, the factors influencing the number of publications on PM are not solely the prevalence of the disease.

**Table 4:** The population-based prevalence of myopic retinopathy and the number of publications since 2020.

**Country/ region**	**Study (*n*)**	**Age**	Prevalence of myopic retinopathy^*^	**Number of publications**
China	Beijing eye study (4,139)	≥40	3.7%	294
Japan	Hisayama study (1,892)	≥40	1.7%	239
Taiwan	Shihpai eye study (1,058)	≥65	3.0%	73
Australia	Blue mountains eye study (3,653)	≥49	1.2%	63

* The definition of myopic retinopathy in various studies is not totally the same.

The number of publications by country or region is usually associated with the interest in and knowledge of a certain field. The global number of publications related to PM has continued to increase since the millennium, with the greatest growth occurring in 2021 (increased by 48 publications compared to 2020), in which China contributed 70.8% of the global growth (34 publications). This increasing trend might have benefited from the state policy of prevention and control of myopia in China, which has been set in recent years (13). Therefore, not only research interest but also administrative encouragement was an important motivation for PM research. State policy can create an encouraging environment for researchers with adequate funding, advanced techniques, and equipment, which are essential for conducting relevant studies.

As PM is a global healthcare challenge, international collaboration has become a mainstream research pattern. In the present study, the USA had the most international collaboration (total link strength of 306 in VOSviewer), followed by China (total link strength of 236), Germany (total link strength of 227), Singapore (total link strength of 213), and Japan (total link strength of 206) (Supplementary Figure 1). The USA is also the second-most productive country, in which authors published ~51% of studies from at least two countries or regions. It can be speculated that the USA was the most productive country. Collaboration and exchange are important in the academic community, and the USA performs well in international research. Although none of the top 10 authors hailed from the USA, the country had the most citations (9,395 total citations) and the highest H-index (53), indicating that the USA's research had a relatively high quality with good international collaboration and communication.

Therefore, not only is the burden of PM on patients, local economics, and public health a significant determinant of promoting PM research, but state policy and international collaboration are also essential factors contributing to global PM research.

We used the metric of the H-index, which has been widely used, to evaluate a country/area's productivity and citation impact of publications. However, this metric has some limitations (14). We did not evaluate the number of authors in an article or a specific author's position in an article (15), which could result in the distribution of citations from an article to all authors equally. Similar to the distribution of authors, the H-index of a country or region does not consider that the contributions from each country or region in the research were usually heterogeneous, and the equal distribution of citations of a publication to all countries and regions might be unjustified. Moreover, this metric does not differentiate between different cited sections of a publication (e.g., results or discussion, etc.) or between different types of publications (e.g., a review or original research). Additionally, this metric counts self-citations, and some intentional citations might have influenced it; for example, citing the publications of potential reviewers or editors (16, 17).

We also investigated the associations between HDI and publications; all of the top 10 authors came from countries with very high HDI. In addition to China, countries with very high HDI contributed the most publications. Patients from medium- and low-HDI countries and regions also suffer from PM; however, the number and influence of publications from these countries and regions need to be improved due to financial-, technical-, and equipment-related restrictions. The application of advanced technology, such as artificial intelligence, telemedicine, and advanced communication technology, might enhance the diagnosis, screening, and regular examinations in these underdeveloped areas.

### Publication tendency in PM research

We analyzed the top papers with the most citations; 80% of the publications were published in clinical ophthalmology journals, indicating that PM research mainly focuses on clinical medicine rather than basic medical research. Similarly, most publications belong to the WOS category of ophthalmology (82.86%). The analysis revealed that this research field involves more clinicians than scientists and also explained the phenomenon that many PM-related manifestations were noticed by applying multimodal imaging methods, but their pathogenesis was not fully understood, such as dome-shaped maculopathy. In the future, greater efforts should be made to investigate the pathogenesis of PM using animal models.

In PM research, the RRI metric has maintained growth from 0.0018% in 2001 to 0.0044% in 2021, and the number of publications has also increased from 24 in 2000 to 156 in 2021. These results indicate a great interest in PM raised in the medical community, which also conforms with the increasing worldwide prevalence of PM (Figure 1B).

The study trends based on keywords can be considered indicators of basic and clinical research directions. Based on the analysis of keywords in the current study, most studies on PM correlate with epidemiology and risk factors, treatments, lesions, and abnormalities. The color-coded average time of appearance map of co-occurrence keywords showed that the research hotspots transformed from treatment for mCNV to epidemiologic and public health studies and then to more manifestations of PM and their treatments. In more detail (Supplementary Figure 4), we noticed that besides the keywords “myopic, high myopic, and pathologic myopia,” the keywords of “photodynamic therapy, ranibizumab, (myopic) choroidal neovascularization” (year of the median from 2006 to 2014), and “optical coherence tomography, myopic maculopathy, myopic traction maculopathy” (year of the median from 2013 to 2017) had a frequency no < 50, which indicates that the main interest of researches focused on treatment for mCNV, followed by PM-related maculopathy and its imaging findings. The latest hotspot keyword was “deep learning,” revealing a novel research direction. Automated diagnosis, screening, and regular examinations with artificial intelligence and advanced communication technology might enhance teleophthalmology and improve practice patterns in these underdeveloped areas.

The changes in keywords (Figures 3A, 4B) in various periods indicate increasing and emerging research themes in the PM field. Based on the bibliometric analysis, especially the keyword and research hotspots analysis, the following themes have reported the latest outcomes.

### Multimodal imaging and pathologic manifestations

Since the millennium, optical coherence tomography (OCT), optical coherence tomography angiography (OCTA), magnetic resonance imaging (MRI), and other imaging methods have been applied in clinical practice, enabling researchers to evaluate pathologic myopic alterations and detect myopic complications (18). For example, current ultrawide-field imaging can show peripheral retinal abnormalities, and swept-source OCT can exhibit vitreous and retinal changes, such as the formation of posterior vitreous detachment, paravascular cystic lesions, and paravascular lamellar holes (19, 20).

#### Posterior staphyloma

Posterior staphyloma is defined as an outpouching of the eye wall with a radius of curvature less than the surrounding curvature of the eye wall (21). However, its formation is not yet fully understood. Currently, ultrawide-field OCT, ultrawide-field photography and MRI can visualize and evaluate the structure of staphyloma and its complications (21–23). Relevant complications include tractional maculopathy (such as retinoschisis), lacquer cracks, mCNV, and chorioretinal atrophy. Traditionally, posterior staphyloma was divided into 10 subtypes by Curtin (24). Recently, five types of posterior staphylomas have been proposed using MRI by Ohno-Matsui (21). Compared with MRI, ultrawide-field OCT provides visualization of retinal tissues simultaneously with scleral changes (usually when the choroid is not too thick, based on our experience).

#### Myopic choroidal neovascularization

Based on the analysis of keywords, we can observe that mCNV has always been the focus of PM research. Approximately 5.2–11.3% of eyes with PM develop mCNV (25). Ninety percent of eyes with PM progress to legal blindness in 10 years without treatment (26). Ischemia and breaks in Bruch's membrane may contribute to the development of mCNV (27, 28). In addition to fluorescein angiography (FA) and OCT, which can determine the activity of mCNV, non-invasive OCTA can demonstrate neovascularization volumetrically and can be performed repeatedly during follow-up. Anti-vascular endothelial growth factor (VEGF) agents are the first-line therapy (29–31).

#### Myopic maculopathy

According to several population-based studies, the frequency of myopic maculopathy varies from 1.2 to 3.8% (32–35). The classification of myopic maculopathy in PM was established using fundus photography (FP) and OCT images (Supplementary Table 2) (36–40). Myopic traction maculopathy can be evaluated using OCT for macular status, as well as surgical indications (41). However, these classification systems do not include some manifestations, such as the dome-shaped macula.

#### Dome-shaped maculopathy

Dome-shaped maculopathy refers to an inward convex protrusion of the macula within the posterior concavity of the eye that is mainly visualized using OCT and MRI (42, 43). The alteration can be detected only in horizontal scanning images, only in vertical images, or in both horizontal and vertical images because of the morphologic pattern of the round dome (43, 44). However, domes in children only appear in vertical OCT scanning images (45). Dome-shaped maculopathy can be identified quantitatively as a macular inward bulge height of more than 50 μm in the most convex image (46). Serous retinal detachment was detected in 2–67% of eyes with dome-shaped maculopathy (42, 46–49), and CNV was observed in 41.2% of the affected eyes (46). A bulge height of more than 400 μm is purportedly associated with decreased visual acuity, serous detachment, and greater retinal pigment epithelial atrophy (50). However, the mechanism of dome formation remains unclear.

#### Myopic glaucoma-like optic neuropathy

In PM, expansion, tilting, torsion, and effacement of the optic nerve head had been noticed, which resembled glaucoma-like neuropathy with loss of the neuroretinal rim (51). However, the irregularity of the optic nerve head shape and macula makes it challenging to determine the neuroretinal rim and to evaluate the thickness of the retinal nerve fiber layers and ganglion cell layers using OCT (52, 53). Fortunately, OCTA might be helpful in the future to differentiate myopia from glaucoma by exploring vascular changes under various conditions (54).

### Impact on public health and management

The global prevalence of PM-related visual impairment is estimated to rise from 0.1% in 2020 to 0.6% in 2050 (55); therefore, the public health burden and impact on the quality of life will likely increase. PM affects patients' reading, mobility, and emotional wellbeing (56). For PM patients who suffer from visual loss or low vision, care and rehabilitation with adaptive technologies are recommended (57, 58).

Patients with myopia are usually unaware that the development of pathological complications might result in irreversible vision impairment (59). Public education campaigns on the increased risk of PM and regular eye examinations are urgently needed, particularly for high-risk individuals. Myopia control and prevention programs, national-level policies, and healthcare providers should be available to PM patients to provide timely medical care (60–63). Moreover, more research was performed on adult subjects (808 publications involving adults vs. 56 publications involving juveniles), and more research on juvenile patients is expected.

Increasing outdoor time (at least 3 h per day) and decreasing near-work activities are beneficial to the prevention and control of myopia (61, 64–66), which also implicitly controls the progression of simple myopia on PM. Optical aids with myopia control properties, including orthokeratology and soft multifocal lenses, can be used in children with myopia (67–69). Atropine and pirenzepine are alternative interventions for myopic control (70–74). Regular measurement of refraction and axial length helps monitor the progression of PM.

### Pathogenesis mechanism

Both environmental and genetic factors contribute to the pathogenesis of PM, and the sclera has profound effects on the development of PM. Environmental factors include educational stress, economic level, outdoor time, and near-work time and intensity. Regarding genetic factors, *CCDC102B* is a susceptibility gene for myopic maculopathy in the Japanese population (75). However, other studies have not found an association between candidate genes and PM.

Animal models with PM features have mainly been established using mice, chicks, and monkeys (76–79). However, no animal models precisely matched the characteristic patterns of PM in human eyes. Therefore, extrapolation from animal models to human beings needs to be done cautiously, and mimicking the formation of PM in human eyes in developing animal models is not promising.

Scleral remolding plays an important role in the pathogenesis of PM (80). Pathologic visual stimulation influences choroidal blood and initiates scleral hypoxia, resulting in the development of myopia and axial elongation (81–83). However, its pathogenesis at the molecular level remains largely unknown.

The sclera has been a treatment target for PM, and several surgical approaches have been reported (84–96). However, no sclera-targeted treatment regimens have been proven safe and effective for the long-term management of PM.

### Artificial intelligence and future direction

Deep learning, as the main component of artificial intelligence, has a great ability to manipulate multiple-dimensional data and perform complex tasks on medical data. Several studies have investigated the diagnostic performance of deep-learning models for identifying PM based on fundus photos or OCT images and have achieved an AUC of more than 0.95 (97–100). Other studies have used deep-learning methods to automatically detect PM-related lesions, such as myopic maculopathy (101–104). However, the performance of these models in real-world clinical practice and population-based screening remains unclear and requires further validation.

Moreover, three directions require further research. First, a deep-learning approach based on multimodal imaging for diagnosing PM and detecting lesions has not yet been studied. The latest ATN classification system comprehensively evaluates myopic maculopathy based on fundus photographs and OCT images (105, 106). Deep-learning methods based on bimodal or multimodal imaging can provide a more precise evaluation of PM. Second, PM is a disease requiring timely diagnosis and regular checks and examinations, which consume large amounts of medical resources unavailable in many underdeveloped countries and regions. Therefore, teleophthalmology systems with embedded automated deep-learning models for PM diagnosis and follow-up could help solve this dilemma in the real world. Third, deep learning in PM may serve additional tasks, including designing treatment regimens and predicting the prognosis of PM.

### Strengths and limitations

The current study was the first bibliometric analysis of PM based on publications since 2000, reflecting the latest updates in this field. Data were extracted from the authoritative WOS Core Collection, and VOSviewer and Biblioshiny were used for bibliometric analysis. However, this study has several limitations. The nature of selection bias existed in the methods; only papers published in authoritative and influential journals that were listed in the WOS Core Collection were included in our analysis, and publications from other databases such as Medline and Scopus were not included. Medline and Scopus did not provide complete records of citations as the WOS Core Collection did. WOS provides better accuracy in document type assignment than Scopus and more comprehensive citation data than Medline (107, 108). Therefore, the WOS database is the most commonly used reference database for bibliometric analysis.

Moreover, no perfect and comprehensive metrics exist for analyzing and predicting future trends in PM research, and the drawbacks of the H-index have been explained. The metric of impact factor is also not perfect. It is only calculated for journals by Clarivate, and this metric is not available for authors or institutions. A comprehensive and consistent metric to evaluate academic influence for authors, institutions, journals, and countries is always needed and under investigation. Furthermore, although we evaluated the number of publications each author participated in, we did not identify the author's positions in individual publications. However, the author's position in an article does not necessarily correlate with their specific contribution to PM research.

## Conclusion

This study comprehensively analyzed published research on PM since the millennium and presents the current status of mainstream studies on PM. PM is a topic of interest for both scientific and clinical research. China, the USA, and Japan contributed the greatest number of publications; the journals, authors, and institutions that published the most relevant literature also came from these three countries. More pathologic changes in the macula have been observed using multimodal imaging methods, and their pathogenesis is under investigation. With the increasing prevalence of PM, interventions for PM have become a public health issue and a research hotspot. Combined with the latest technology, including artificial intelligence, automated diagnosis, and screening of PM is a novel field. Taken together, our results should help researchers understand the current status and provide future directions.

## Data availability statement

The original contributions presented in the study are included in the article/Supplementary material, further inquiries can be directed to the corresponding author.

## Author contributions

JY: design, definition of intellectual content, data acquisition, data analysis, manuscript preparation, and manuscript editing. SW: design, definition of intellectual content, data acquisition, data analysis, funding, manuscript preparation, and manuscript editing. CZ: data analysis and manuscript review. All authors contributed to the article and approved the submitted version.

## Funding

This work was funded by National High Level Hospital Clinical Research Funding (BJ-2021-208) and National Natural Science Foundation of China (82200730).

## Conflict of interest

The authors declare that the research was conducted in the absence of any commercial or financial relationships that could be construed as a potential conflict of interest.

## Publisher's note

All claims expressed in this article are solely those of the authors and do not necessarily represent those of their affiliated organizations, or those of the publisher, the editors and the reviewers. Any product that may be evaluated in this article, or claim that may be made by its manufacturer, is not guaranteed or endorsed by the publisher.

## Supplementary material

Supplementary Figure 1 The co-occurrence map of 34 countries and regions, which showed the international collaboration among countries/regions.

Supplementary Figure 2 The distribution of publication year for publications of various citation frequency **(A)**. High frequency: more than 100 citations; medium frequency: more than 50 citations and <100 citations; low frequency <50 citations. The heatmaps of high citation frequency (more than 100 citations) group **(B)**, medium frequency (more than 50 citations and <100 citations) group **(C)**, and low frequency (<50 citations) group **(D)** in each year, respectively. Every row in the heatmap represents a publication. The color represents the total citation number in each year (x axis).

Supplementary Figure 3 The co-occurrence map of scholars who published papers of pathologic myopia, which showed the cooperation among researchers.

Supplementary Figure 4 The top keywords with strongest burst in pathologic myopia research since 2,000 based on the authors' keywords lists. On the base timeline, the location of the circles represents the median year of the duration when keywords were used frequently, and the size of the circles represents the frequency. The blue segments represent the first quartile time point to the third quartile time point of the duration when keywords were used frequently.

## Abbreviations

FA: fluorescein angiography
FP: fundus photograph
HDI: human development index
mCNV: myopic choroidal neovascularization
MRI: magnetic resonance imaging
OCT: optical coherence tomography
OCTA: optical coherence tomography angiography
PM: pathologic myopia
RRI: relative research interest
VEGF: vascular endothelial growth factor
WOS: web of science.
