# Supplementary figures and images for: Global trends and frontiers of research on pathologic myopia since the millennium: A bibliometric analysis
Source: Front Public Health. 2022 Dec 6;10:1047787. doi: 10.3389/fpubh.2022.1047787 (PMC9763585; doi:10.3389/fpubh.2022.1047787)

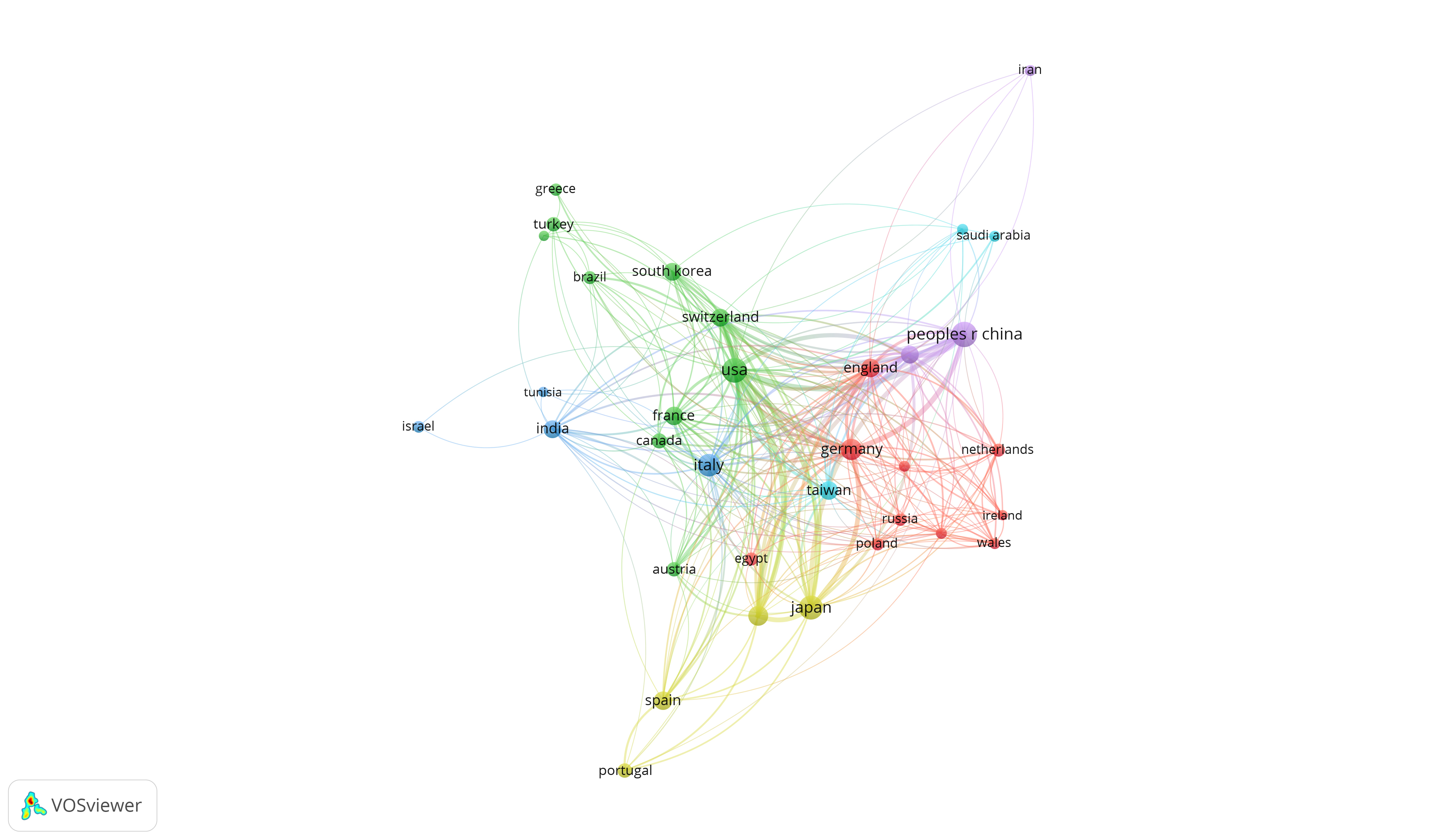

Supplement: Supplementary Figure 1 — The co-occurrence map of 34 countries and regions, which showed the international collaboration among countries/regions. [file Image_1.JPEG]

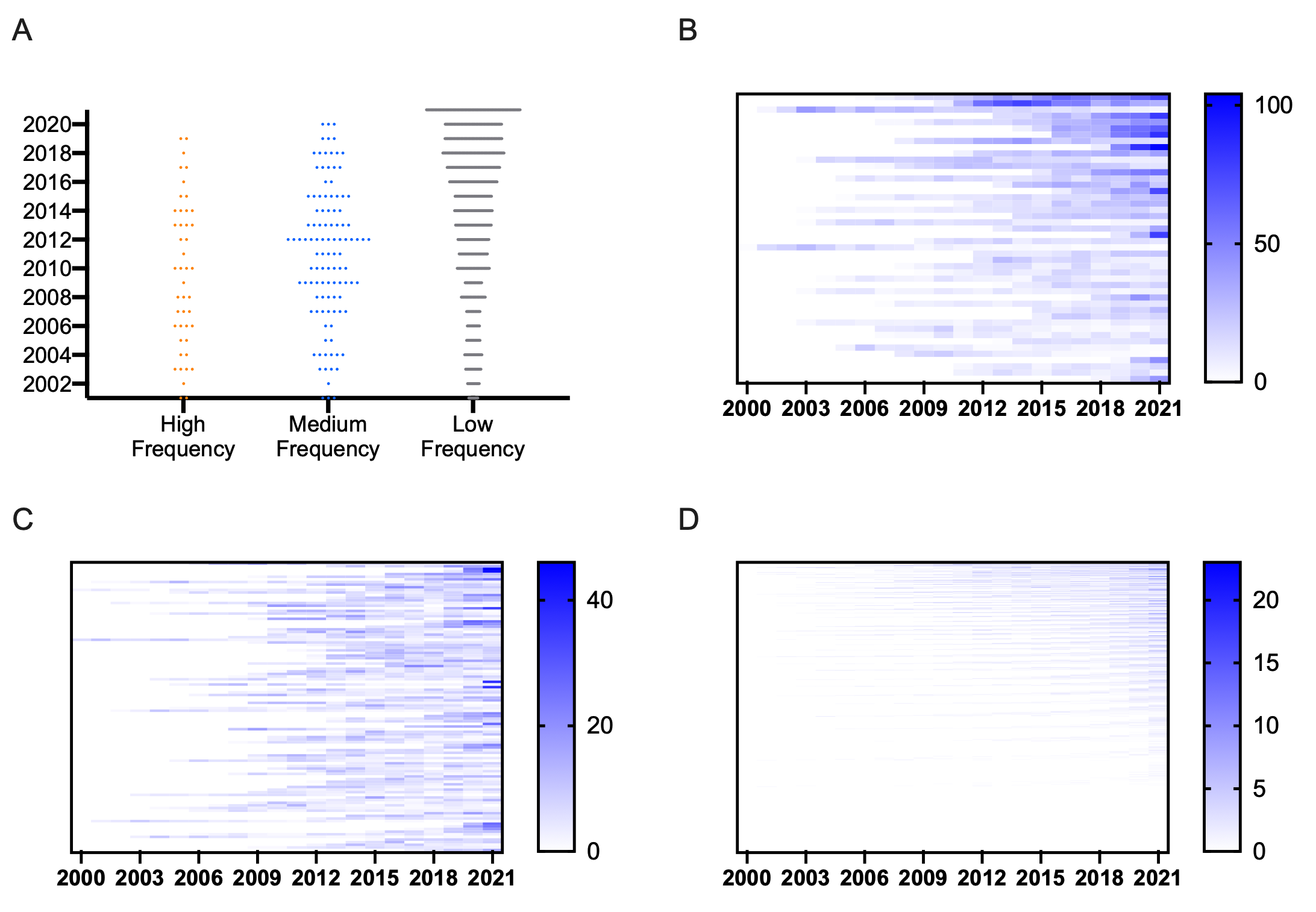

Supplement: Supplementary Figure 2 — The distribution of publication year for publications of various citation frequency (A). High frequency: more than 100 citations; medium frequency: more than 50 citations and <100 citations; low frequency <50 citations. The heatmaps of high citation frequency (more than 100 citations) group (B), medium frequency (more than 50 citations and <100 citations) group (C), and low frequency (<50 citations) group (D) in each year, respectively. Every row in the heatmap represents a publication. The color represents the total citation number in each year (x axis). [file Image_2.TIFF]

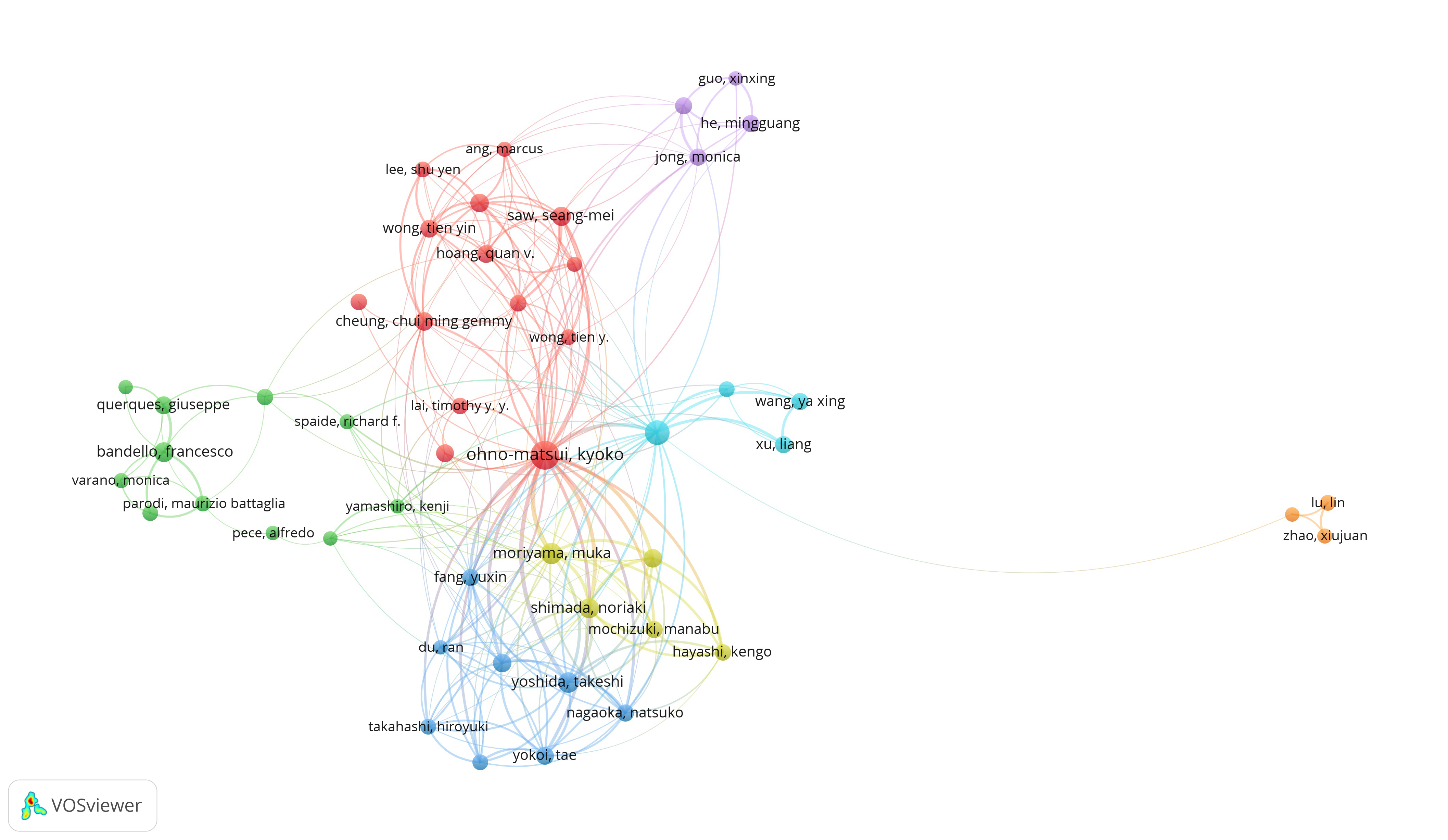

Supplement: Supplementary Figure 3 — The co-occurrence map of scholars who published papers of pathologic myopia, which showed the cooperation among researchers. [file Image_3.JPEG]

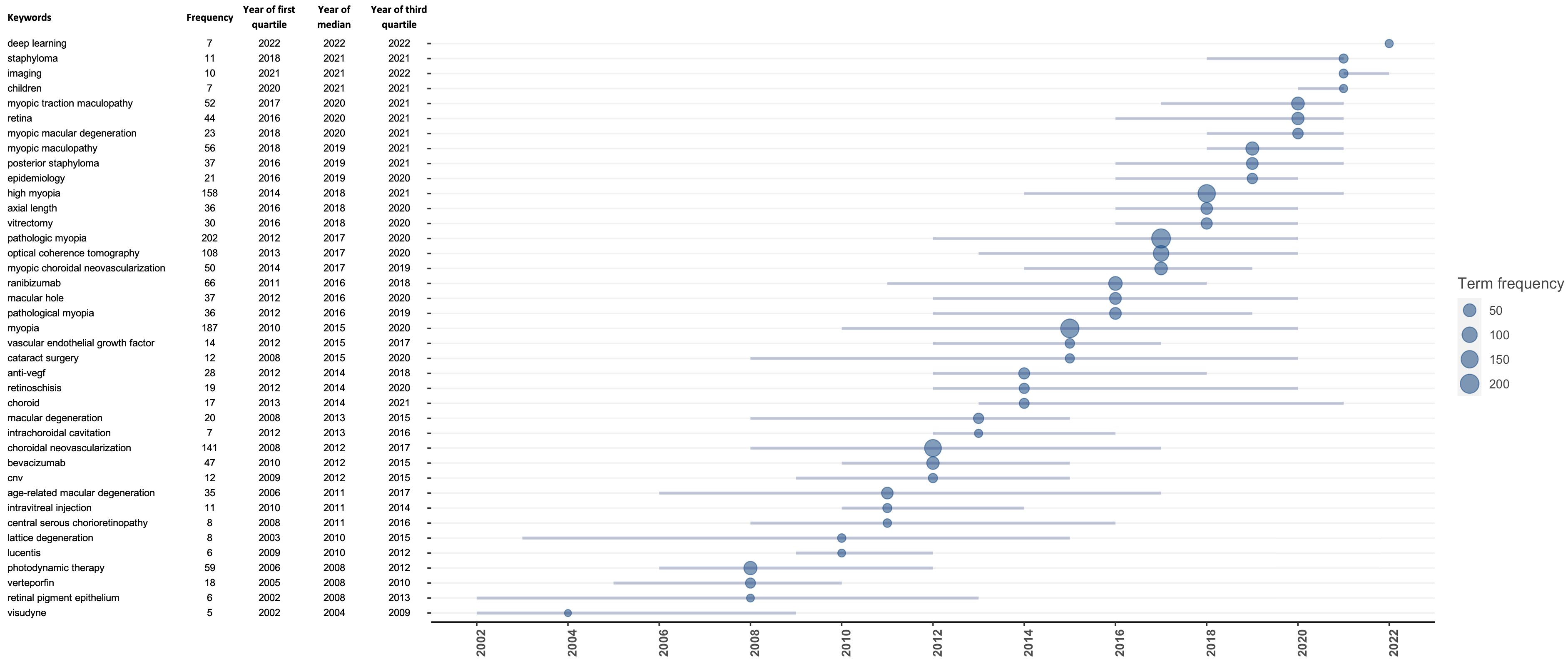

Supplement: Supplementary Figure 4 — The top keywords with strongest burst in pathologic myopia research since 2,000 based on the authors' keywords lists. On the base timeline, the location of the circles represents the median year of the duration when keywords were used frequently, and the size of the circles represents the frequency. The blue segments represent the first quartile time point to the third quartile time point of the duration when keywords were used frequently. [file Image_4.PNG]
