# Supplementary material for: Global trends and frontiers of research on pathologic myopia since the millennium: A bibliometric analysis
Source: Front Public Health. 2022 Dec 6;10:1047787. doi: 10.3389/fpubh.2022.1047787 (PMC9763585; doi:10.3389/fpubh.2022.1047787)
Supplement: Supplementary file 5 [file Data_Sheet_1.PDF]

Supplementary Table 1. The number of total citations of the top 20 countries/regions.

Countries/regions	Number of citations
USA	9395
Japan	6999
China	5559
Singapore	4048
Germany	3915
Italy	3297
England	2817
Australia	2742
Switzerland	2242
Spain	1936
Taiwan, China	1650
France	1547
Canada	958
South Korea	950
Austria	799
Portugal	663
India	572
Brazil	179
Turkey	99
Egypt	85

Supplementary Table 2. Various classification systems for myopic maculopathy.

ATN classification[1]			Myopic foveoschisis classification[3]	Myopic foveoschisis classification[4]	Myopic maculopathy classification[5]
Atrophic component/ maculopathy atrophy classification	Tractional component	Neovascular component			
**A0:** No myopic retinal lesions<br>**A1:** Tessellated fundus only<br>**A2:** Diffuse chorioretinal atrophy<br>**A3:** Patchy chorioretinal atrophy<br>**A4:** Complete	**T0:** no macular schisis<br>**T1:** Inner or outer foveoschisis<br>**T2:** Inner + outer foveoschisis<br>**T3:** Foveal detachment<br>**T4:** Full-thickness	**N0:** No myopic choroidal neovascularization<br>**N1:** Macular lacquer cracks<br>**N2a:** Active choroidal neovascularization<br>**N2s:** Scar/Fuch's	**S0:** Absent<br>**S1:** Extrafoveal<br>**S2:** Foveal only<br>**S3:** Foveal but not entire macula<br>**S4:** Entire macula	**Inner layers:** Inner plexiform layer + ganglion cell layer + retinal fiber layer<br>**Outer layers:** Outer plexiform layer + inner plexiform layer	**Ia:** Peripapillary choroidal thinning<br>**Ib:** Macular choroidal thinning<br>**Plus sign:** Linear Bruch's membrane defects<br>**II:** Extrafoveal Bruch's membrane defects<br>**Plus sign:** Myopic choroidal

macular atrophy **Plus signs in classification for only maculopathy atrophy**[2]**:** Lacquer cracks, Fuch’s spot, and myopic choroidal neovascularization	macular hole **T5:** macular hole + retinal detachment	spot		**Inner + outer layers**	neovascularization **III:** Foveal Bruch’s membrane defects **IIIb:** choroidal neovascularization -related **IIIa:** patchy related **Plus sign:** Macular traction maculopathy, dome-shaped macular and macular ridge

1. Ruiz-Medrano J, Montero JA, Flores-Moreno I, et al. Myopic maculopathy: Current status and proposal for a new classification and grading system (ATN). Prog Retin Eye Res 2019;69:80-115. doi: 10.1016/j.preteyeres.2018.10.005 [published Online First: 2018/11/06]

2. Ohno-Matsui K, Kawasaki R, Jonas JB, et al. International photographic classification and grading system for myopic maculopathy. Am J Ophthalmol 2015;159(5):877-83.e7. doi: 10.1016/j.ajo.2015.01.022 [published Online First: 2015/01/31]

3. Shimada N, Tanaka Y, Tokoro T, et al. Natural course of myopic traction maculopathy and factors associated with progression or resolution. Am J Ophthalmol 2013;156(5):948-57.e1. doi: 10.1016/j.ajo.2013.06.031 [published Online First: 2013/08/27]

4. Fujimoto M, Hangai M, Suda K, et al. Features associated with foveal retinal detachment in myopic macular retinoschisis. Am J Ophthalmol 2010;150(6):863-70. doi: 10.1016/j.ajo.2010.06.023 [published Online First: 2010/10/19]

5. Ohno-Matsui K, Wu PC, Yamashiro K, et al. IMI Pathologic Myopia. Invest Ophthalmol Vis Sci 2021;62(5):5. doi: 10.1167/iovs.62.5.5 [published Online First: 2021/04/29]
